# Bilateral intracochlear schwannomas: histopathological confirmation and outcomes following tumour removal and cochlear implantation with lateral wall electrodes

**DOI:** 10.1007/s00106-023-01379-7

**Published:** 2023-10-30

**Authors:** Mark E. Quick, Shannon Withers, Stefan K. Plontke, Ronel Chester-Browne, Jafri Kuthubutheen

**Affiliations:** 1 Department of Otolaryngology, Sir Charles Gairdner Hospital, Hospital Avenue, 6009 Nedlands, Perth, Western Australia Australia; 2 Division of Surgery, Medical School, University of Western Australia, Nedlands, Perth, Western Australia Australia; 3 Department of Otorhinolaryngology, Head and Neck Surgery, Martin Luther University Halle-Wittenberg, Halle (Saale), Germany; 4 Ear Science Implant Clinic, Perth, Western Australia Australia

**Keywords:** Acoustic neuroma, Cochlea, Cochlear implant, Inner ear, Intralabyrinthine, Akustikusneurinom, Cochlea, Cochleaimplantat, Innenohr, Intralabyrinthär

## Abstract

Intracochlear schwannomas (ICS) are very rare benign tumours of the inner ear. We present histopathological proof of the extremely rare bilateral occurrence of intracochlear schwannomas with negative blood genetic testing for neurofibromatosis type 2 (NF2). Bilateral schwannomas are typically associated with the condition NF2 and this case is presumed to have either mosaicism for NF2 or sporadic development of bilateral tumours. For progressive bilateral tumour growth and associated profound hearing loss, surgical intervention via partial cochleoectomy, tumour removal, preservation of the modiolus, and simultaneous cochlear implantation with lateral wall electrode carrier with basal double electrode contacts was performed. The right side was operated on first with a 14-month gap between each side. The hearing in aided speech recognition for consonant-nucleus-consonant (CNC) phonemes in quiet improved from 57% to 83% 12 months after bilateral cochlear implantation (CI). Bilateral intracochlear schwannomas in non-NF2 patients are extremely rare but should be considered in cases of progressive bilateral hearing loss. Successful tumour removal and cochlear implantation utilizing a lateral wall electrode is possible and can achieve good hearing outcomes.

## Abstract (de)

Intracochleäre Schwannome (ICS) sind sehr seltene benigne Tumoren des Innenohrs. In der vorliegenden Arbeit wird die histopathologische Bestätigung des extrem seltenen bilateralen Vorkommens von intracochleären Schwannomen mit negativem genetischem Bluttest auf Neurofibromatose 2 (NF2) vorgestellt. Bilaterale Schwannome sind typischerweise mit NF2 assoziiert, und bei dem vorliegenden Fall wird vermutet, dass entweder ein Mosaik für NF2 oder die sporadische Entwicklung von bilateralen Tumoren besteht. Bei progressivem bilateralem Tumorwachstum und damit einhergehender hochgradiger Schwerhörigkeit wurde eine chirurgische Intervention mit partieller Cochleoektomie, Tumorentfernung, Erhalt des Modiolus und gleichzeitiger Cochleaimplantation unter Einsatz eines „lateral wall“-Elektrodenträgers mit basal doppelten Elektrodenkontakten durchgeführt. Zuerst wurde die rechte Seite operiert und mit einem Abstand von 14 Monaten die zweite Seite. Das Sprachverstehen für Konsonant-Nucleus-Konsonant(CNC)-Phoneme in Ruhe verbesserte sich 12 Monate nach bilateraler Cochleaimplantation von 57 auf 83%. Bilaterale intracochleäre Schwannome bei Nicht-NF2-Patienten sind extrem selten, aber sollten in Fällen mit progressiver bilateraler Schwerhörigkeit in Erwägung gezogen werden. Die erfolgreiche Tumorentfernung und Cochleaimplantation unter Verwendung von „lateral wall“-Elektrodenträgern ist möglich und kann zu guten Hörergebnissen führen

Vestibular schwannomas (VS) are common benign intracranial tumours that usually arise from the Schwann cell sheath of the inferior or superior vestibular nerves [1]. They arise predominantly in the internal auditory canal (IAC) and can expand into the cerebellopontine angle (CPA). A type of schwannoma of the eighth cranial nerve primarily arising in the inner ear is known as *inner ear schwannoma* (IES) or *intralabyrinthine schwannoma *(ILS; [2–4]). These tumours are also thought to arise from Schwann cells associated with the terminal branches of the cochlear nerve or vestibular nerve.

With an incidence of 0.81 per 100,000 person-years, IES are a very rare subtype of schwannomas of the eighth cranial nerve [5]. They were described as early as in 1917 during inner ear autopsies [6]. These tumours can remain undetected for several years but with the development of high-resolution gadolinium enhanced magnetic resonance imaging (MRI) the frequency of diagnosis of these lesions has increased. An ILS classification system based on MRI images was first proposed by Kennedy et al. in 2004 [4]. This classification system divides these tumours into seven entities including intracochlear, intravestibular, intravestibulocochlear, transmodiolar, transmacular, transotic, and tympanolabyrinthine schwannomas. Additional subsites were added by van Abel et al. (2013) and include translabyrinthine, involving the cerebello-pontine angle (CPA), and unspecified [3].

The most common subsite for IES is intracochlear and these tumours are therefore known as *intracochlear schwannomas* (ICS). Patients with ICS can present with hearing loss (in 94.5% of cases), more rarely sudden hearing loss (29.6%), tinnitus (67.6%), and vertigo (50.7%) [2]. Patients may also be asymptomatic with very small tumours [7]. The published data on tumour position correlating with symptom presentation are mixed. A systematic review published by Elias et al. [8] described a strong correlation whilst Salzman et al. [9] showed no reported link within their series. As ICS are a benign condition, the majority of patients with serviceable hearing remain under clinical observation, undergoing serial imaging every 6 months for the first year and then yearly up to 5 years with regular audiological testing. Intervention is typically recommended for patients with tumour growth and progressive hearing loss (i.e., non-serviceable hearing) or intractable symptoms such as vertigo. Both surgery and stereotactic radiation are treatment options with the latter only for select cases such as in an older or inoperable patients.

Cochlear implantation (CI) with penetration of the ICS and no tumour removal has also been described in the literature [10, 11]. Complications including resistance on implant insertion or tip “roll-over” with the use of a second implant, however, have been reported [10, 11] and tumour growth in these cases is not stopped. Simultaneous ICS tumour removal and CI placement is challenging as the cochlear capsule is inevitably destroyed requiring reconstruction. However, good hearing outcome with CI and preservation of vestibular function can be achieved despite such major trauma to the cochlea [12, 13].

Withers et al. (2020) previously published an extremely rare case of bilateral ICS established on clinical and radiological MRI findings [14]. Here we present histopathological proof of the presence of bilateral ICS in a patient without neurofibromatosis type 2 (NF2), and the audiological outcome after simultaneous tumour removal and CI first in one and then in the contralateral ear.

## Case presentation

### History and clinical presentation

**Fig. 1:**
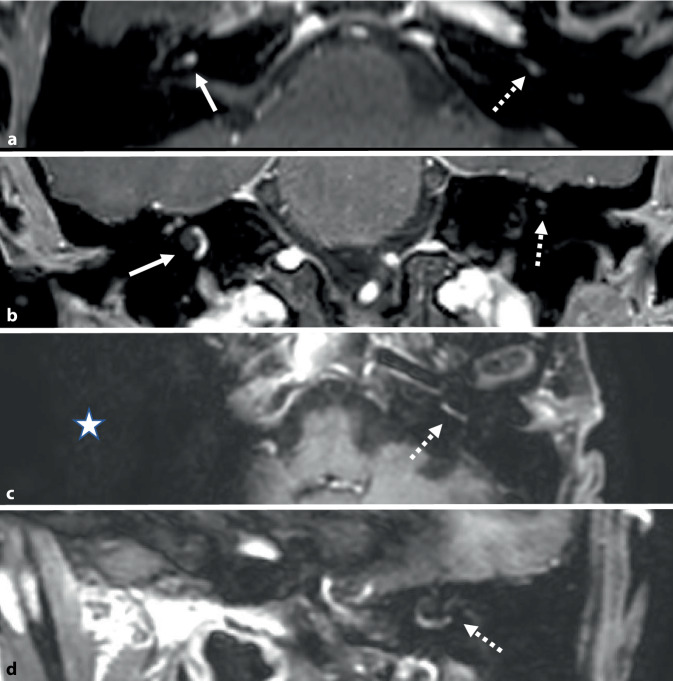
Intracochlear schwannomas (ICS) identified on axial and coronal gadolinium (Gd)-enhanced T1-weighted magnetic resonance imaging (MRI; Philips DRIVE T3 MRI scanner). Enhancing right ICS (*solid arrows*) on axial (**a**) and coronal (**b**) and early small left ICS (*dashed arrows*) prior to right-sided surgical intervention. Progressive growth of left-sided ICS (*dashed arrows*) on axial (**c**) and coronal (**d**) repeat MRI after right-sided surgery with artifact (*star*) from cochlear implant

**Fig. 2:**
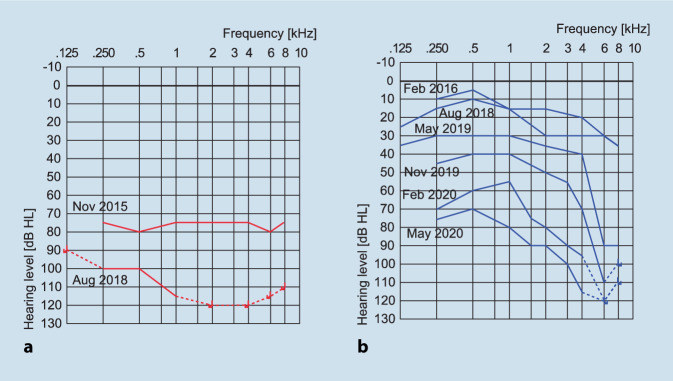
Time course of pure tone audiogram thresholds (only air conduction shown) for the right (**a**) and left ear (**b**) prior to the respective surgical interventions

A 63-year-old Caucasian female patient initially presented with an 8‑year history of right-sided hearing loss, associated with non-pulsatile tinnitus and two brief episodes of vertigo. The MRI examination demonstrated an enhancing small mass within the cochlea, suggestive of a right ICS. A “wait-and-scan” approach was initially decided with close surveillance. On subsequent serial imaging an early-stage left-sided ICS was also identified (Fig. 1) with both showing progressive growth on repeat imaging over a 32-month period from the initial right-sided tumour diagnosis. Audiometric monitoring tracked deterioration in her hearing over this period to profound sensorineural hearing loss (SNHL) in her right ear (Fig. 2a), after which a multidisciplinary team discussion recommended surgical intervention, with simultaneous tumour resection and CI on the right side. By the time of the intervention, the right ICS measured 8 mm on imaging. At this stage, the left ICS was being monitored with mild-to-moderate down sloping SNHL, which progressively developed to worsening of the hearing along with tumour growth (Figs. 1c, d **and** 2b). A similar surgery was performed on the left ear 14 months after the right-sided surgery including CI for hearing rehabilitation.

### Surgery

**Fig. 3:**
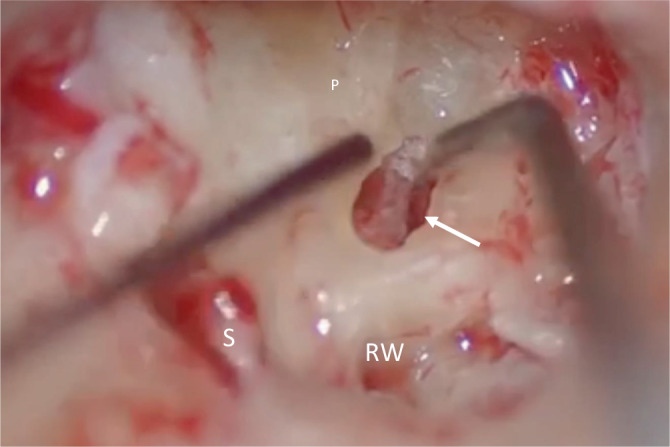
Intra-operative image after subtotal petrosectomy. Initially an opening along the basal turn is drilled with identification of the intracochlear schwannoma (*solid arrow*). *S* stapes, *P* promontory, *RW* round window. *Dark* *lines*: suction tip (*left*) and Shambaugh Ear Hook (*right*)

**Fig. 4:**
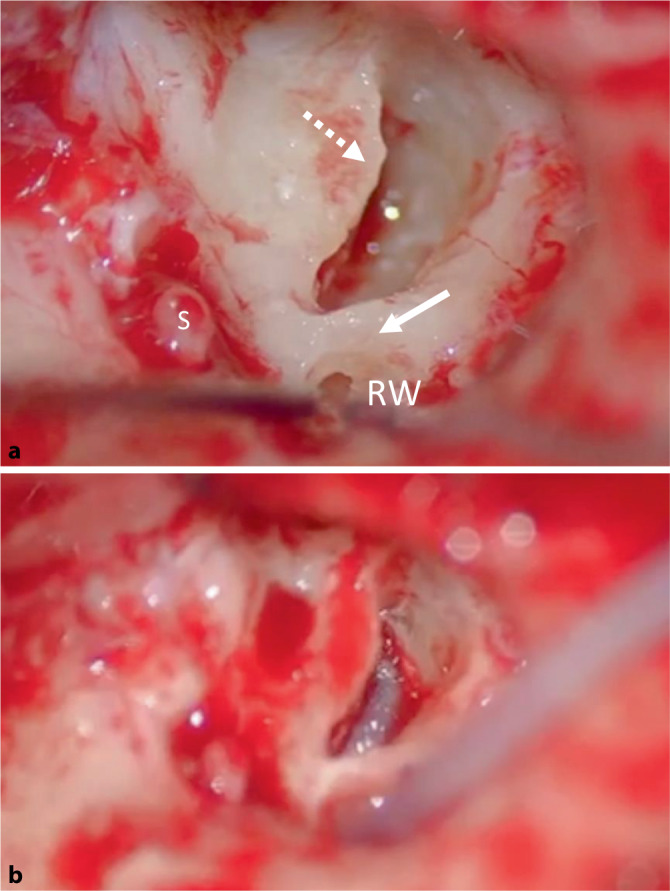
**a** Complete tumour resection after subtotal cochleoectomy (*dashed arrow*) preserving the modiolus and lateral round window arch (*solid arrow*). **b** Standard cochlear insertion technique through the round window and confirmation of insertion along the basal turn. *S* stapes, *RW* round window

A blind sac closure of the external auditory meatus with obliteration of the eustachian tube and canal wall-down mastoidectomy was performed prior to tumour resection with a microscope. On the right side the ICS was identified on opening the round window with tumour extending to the cochlea–carotid junction of the basal turn (Fig. 3). The left tumour extended the entire length of the basal turn from the round window to just medial of the modiolus. Complete bilateral macroscopic clearance of the tumour was achieved using a “push through” technique preserving the entire modiolus. The tumour had not visibly invaded the modiolus, making tumour removal easier in both cases. Each side had a standard 12-electrode channels 24-mm cochlear implant (Mi1200+Medium, MED-EL, Austria) inserted through a preserved round window arch (Fig. 4a,b). The round window arch was preserved to support and assist in holding the electrode in position. A complete insertion was achieved for both operated sides. Standard intra-operative neural response telemetry (NRT) was conducted after cochlear insertion confirming implant function and full response for all electrodes tested. Stabilisation of the cochlear implant with cartilage chips was performed and reconstruction of the lateral wall surrounding the exposed cochlear implant included a combination of tragal cartilage, temporalis fascia and bone pâté with support from fibrin glue (Tisseel, Baxter, IL, USA).

### Histopathology

**Fig. 5:**
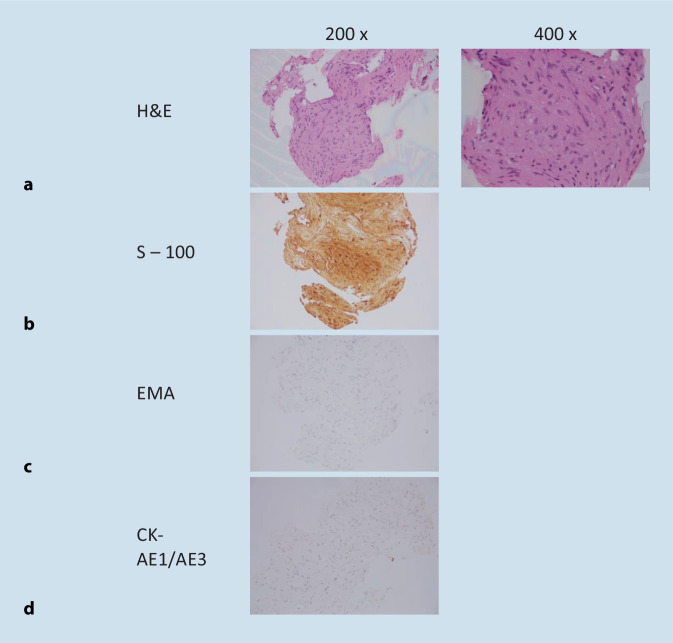
Right-sided intracochlear schwannoma confirmed on histology and immunohistochemistry. **a** Compact spindle shaped cells with Antoni A and Antoni B pattern palisading around a nuclear free area; H&E. **b** Strong positive tumour cells; S‑100. **c** EMA and **d** CK-AE1/AE3 both of tumour cells. *H&E* haematoxylin and eosin stain. *EMA* epithelial membrane antigen

**Fig. 6:**
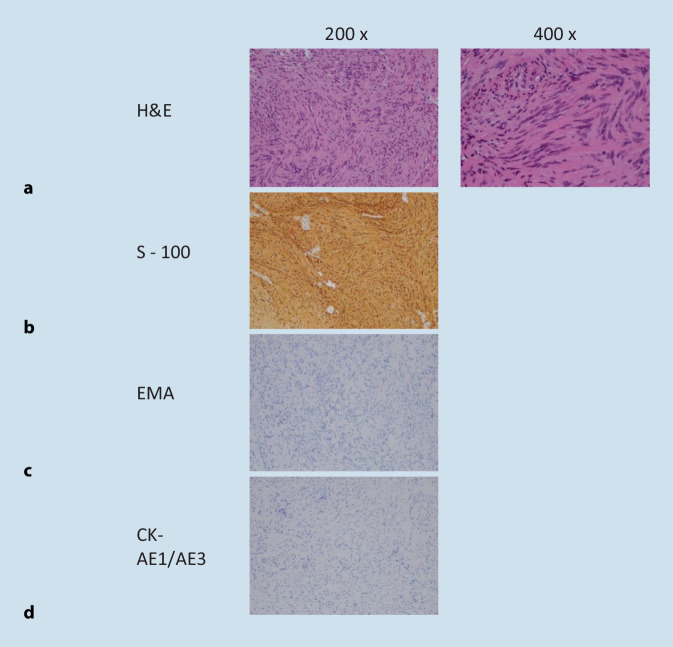
Left-sided intracochlear schwannoma confirmed on histology and immunohistochemistry. **a** Compact spindle-shaped cells with Antoni A and Antoni B pattern palisading around a nuclear-free area; H&E. **b** Strong positive tumour cells; S‑100. **c** EMA and **d** CK-AE1/AE3 both of tumour cells. *H&E,* haematoxylin and eosin stain. *EMA* epithelial membrane antigen

Formal histopathology confirmed the clinical diagnosis of a schwannoma on both sides. Spindle cells and bland nuclei with areas of palisading whirls with scattered blood vessels were identified on microscopic assessment with no atypia or mitotic activity. The tumour was positive for S 100 protein (s-100) and SRY-related HMG-box 10 protein (sox-10), but was negative for epithelial membrane antigen (EMA) and anticytokeratin monoclonal antibodies AE1 and AE3 (AE1/3). This was consistent with a benign schwannoma for both sides (Figs. 5 and 6). As previously reported, the patient tested negative for *NF2* gene mutation in unaffected tissues (blood) using multiplex ligation-dependent probe amplification (MLPA; [14]).

At the routine 8‑week post-operative review, following her second procedure (left ICS removal and cochlear implant insertion), the patient had well-healed surgical sites. She was able to perform well with gait, Rhomberg and Unterberg testing. Formal balance testing showed mild right beating nystagmus with oculomotor testing with Frenzel goggles. She had positive bilateral head impulse and negative Hallpike and log roll testing. Her imbalance subjectively improved with nil balance concerns 6 months after the second procedure. Post-operative testing showed good electrode impedance with no open or short circuits on each side. At the last post-operative review, she had been wearing the left cochlear implant for 16 months and the right one for 24 months.

Aided speech perception (CNC words in quiet for phonemes delivered at 65 dB sound pressure level) indicated excellent hearing bilaterally and a significant binaural benefit (Table 1). The left ear (second implantation) improved from pre-operatively 13% to 77% at 12 months post-operatively. The right ear further improved from 50% at 12 months post-operatively to 73% when retested at 24 months after implantation. Bilateral functional testing showed a mean improvement for binaural aided speech, understanding from 57% before to 83% at 12 months after the second implant. Similar improvement individually and binaural aided was identified with CUNY (City University of New York) sentences. Speech perception in noise indicated limited benefit, which is to be expected, with current ongoing audiological support for mapping variations to better cope in these environments.

**Table 1:** Speech audiometry results: CNC phonemes and CUNY sentences understood at 65 dB SPL relative to the CI surgery of the respective side

	Time point of measurement			
Side	Pre-op	Post-op 6 months	Post-op 12 months	Post-op 24 months
*CNC phonemes*				
*R*	0%	45%	50%	73%
*L*	13%	66%	77%	n.t.
*R* *+* *L*	57% (CI R only)	68%*	83%*	n.t.
*CUNY sentences*				
*R*	0%	31%	85%	93%
*L*	0%	89%	97%	n.t.
*R* *+* *L*	93% (after CI R)	97%*	98%*	n.t.

After CI surgery, aided speech testing in quiet

*CI *cochlear implant, *SPL* sound pressure level, *R* right, *L* left, *CNC* consonant-nucleus-consonant, *CUNY* City University of New York, *n.t.* not tested

*Bilateral testing (R + L) refers to time point after second (left) CI surgery

Due to susceptibility artefacts from bilateral cochlear implants, MRI of the middle ear and membranous labyrinth was not able to assess tumour recurrence; however, macroscopic (surgical microscope) gross tumour clearance without remnant tumour was achieved at the time of surgery. The patient continues to receive routine audiological follow-up and implant assessment.

## Discussion

Intracochlear schwannomas confined exclusively to the cochlea are very rare tumours, let alone occurring bilaterally. This case is—to the best of our knowledge—the first of its kind in the published literature confirming the presence of ICS on histopathology, immunohistochemistry as well as genetic testing. A case published by Nam et al. (2011) revealed bilateral intracochlear schwannomas on post-mortem temporal bone histopathology in NF2 patients [15]. However, the diagnosis of NF2 was based primarily on the occurrence of bilateral schwannomas without genetic testing, with location not a certainty in the diagnostic criteria for NF2 [16]. Early published cases of unilateral ICS were from incidental diagnosis during other surgical procedures or post-mortem autopsy [17–21]. Advances in MRI technology have enabled physicians to detect these tumours within the inner ear at a very small size and therefore at an early stage [4, 9, 21, 22]. Furthermore, MRI has become the gold standard in diagnosis, now enabling differentiation from other conditions including labyrinthitis, haemorrhage and lipoma after serial imaging or additional computed tomography (CT) for identifying labyrinthitis ossificans.

While bilateral ICS in patients with no genetic or clinical features of NF2 appear to be extremely rare, the possible explanations for this include mosaicism for NF2 or sporadic development of bilateral tumour development by chance alone [14]. Previously, lower rates of 20–33% of mosaicism in NF2 were published [23, 24]. However, recent research by Evans et al. has assessed the overall rate of NF2 mosaicism to now be between 50% and 60% [25]. This is in cases where patients presenting with bilateral schwannomas have no affected parent or family history of NF2 and do not return a positive genetic test. Age of onset is a major factor, with approximately 21.7% of patients less than 20 years old predicted to have mosaicism for NF2 compared to 80.7% in those older than 60 [25]. Although mosaicism has been documented in several tumour predisposition syndromes, NF2 appears to have the highest recorded number of de novo mosaicism cases. This higher level of mosaicism previously published, only leaves a small number of cases with unidentified variants or sporadic development.

Once diagnosed, many factors contribute to the management of ICS, including tumour size, location and growth, initial hearing status and deterioration as well as patient factors. Especially patients with serviceable hearing and without vertigo can be monitored by surveillance (serial imaging and audiologic testing) similarly to the management of “classic” VS in the IAC or CPA. However, tumour growth into the vestibule or through the modiolus into the internal auditory canal complicates management, because hearing rehabilitation with a cochlear implant and complete tumour removal is not possible in transmodiolar tumours and vertigo attacks in the patient’s history decrease the chance of preservation of vestibular function after subtotal cochleoectomy [13, 26]. For patients with ICS and unserviceable hearing, surgical tumour removal and rehabilitation of hearing loss with CI should be considered due to favourable outcomes [12, 13, 27].

Since the possibility of hearing rehabilitation after radiation of an intracochlear tumour is unclear, this may be considered for tumours which have invaded critical areas including IAC or cerebellopontine angle.

Improved surgical visibility was achieved in the described case following a blind sac closure and partial cochleoectomy. This was advantageous for sufficient surgical view with the surgical microscope, both for tumour removal and CI placement. Other approaches including combined tympanomastoid and maintaining the external ear canal have been described [27–29]. The use of micro-endoscopes described by Marchioni et al. (2018) is another technique which can be used to aid tumour removal but was not performed in the presented case [30]. Maintenance of the round window bony arch used in the described case is also supported in previously published studies [27, 29]. This technique allowed for the selection of a standard implant array and insertion method with additional implant support when reconstructing the cochlea and reducing the potential dislocation of electrodes. In this patient, a lateral wall electrode array was used because at the time, the cochlear implant receiver stimulator was 3 T MRI compatible. This case demonstrates that good audiological outcomes can be achieved even when lateral wall arrays are used although more extensive cochlear dissection would have necessitated a perimodiolar array to prevent lateralization of the electrode and to promote modiolar hugging.

Good audiological outcomes have been obtained with ICS tumour removal despite partial or even subtotal cochleoectomy [12]. Preservation of the modiolus of the basal turn without tumour involvement influences hearing outcomes [12, 27, 29]. Simultaneous tumour resection and CI insertion is a viable option in managing ICS. The presented case had metachronous bilateral tumour resection and simultaneous cochlear implantation with excellent and stable audiometric outcomes 12–24 months after CI.

## Practical conclusion

- Bilateral intracochlear schwannoma are extremely rare but may occur in non-NF2 patients.
- Patients presenting with such bilateral schwannomas later in life may not have NF2, with the likelihood of mosaicism or chance occurrence present.
- Simultaneous cochlear implant insertion following tumour removal can be performed with successful hearing rehabilitation even when utilizing a lateral wall electrode.

## Declarations

For this article no studies with human participants or animals were performed by any of the authors. All studies mentioned were in accordance with the ethical standards indicated in each case. For images or other information within the manuscript which identify patients, consent was obtained from them and/or their legal guardians.
